# WORKING TOWARD AN AGE-FRIENDLY UINDY: EXAMINING THE PROCESS AS IT UNFOLDS

**DOI:** 10.1093/geroni/igac059.3064

**Published:** 2022-12-20

**Authors:** Lisa Borrero, Heidi Ewen

**Affiliations:** University of Indianapolis, Indianapolis, Indiana, United States; University of Indianapolis, Indianapolis, Indiana, United States

## Abstract

Universities must adapt by expanding their understanding of inclusivity in higher education, acknowledging new aging-related career opportunities for students and their role as destinations for lifelong learning and community engagement for older adults. Accordingly, nearly 100 institutions have committed to age-inclusivity on their campuses by achieving Age-Friendly University (AFU) designation. For those interested in pursuing this designation at their institutions, an important part of the process is learning from one another about strategies used at other campuses that could be tailored to their own needs. With this in mind, this poster presentation will outline the initial strategies and resulting outcomes of the path to AFU designation at the University of Indianapolis (UIndy) via a preliminary “discovery” phase. Using GSA’s Tools for Advancing Age Inclusivity in Higher Education as a guide, AGHE members at the University of Indianapolis are in the beginning stages of their own process, characterized by discussions with various campus stakeholders during the recent spring semester. This “listening tour” has revealed strong support of the AFU principles and an eagerness to be part of an age-inclusive vision. These conversations have identified existing infrastructure and other strengths that support age-inclusivity, as well as current challenges to be managed and new opportunities to pursue. The authors will also describe the next phase of the process, including interviews with older UIndy alumni and retired faculty and staff about engagement with retired community members, as well as subsequent steps, such as working with colleagues to design viable age-friendly initiatives.

